# Mortality of Philadelphia, for October, November and December, 1853, Collated from the Register Kept at the Health Office; with a Summary for the Entire Year

**Published:** 1854-02

**Authors:** Wilson Jewell


*Mortality of Philadelphia, for October, November and December, 1853, collated from the register kept at the Health Office; with a summary for the entire year.* By Wilson Jewell, M. D.

The whole number of deaths, recorded at the Health office, for the 4th quarter of 1853, commencing Oct. 1st, and ending Dec. 31st,—amounting to 92 days, or 13 weeks and one day—has been 2076, equal to 22½ deaths per day, or, to the population, as 1 in every 299½, estimating the same at 415,000.

The rate of deaths for this quarter, has been 18 per cent. less than the average for the three preceding quarters, and, compared with the deaths for the same quarter of 1852, a fraction under.

About 46 per cent., or nearly one half the deaths for the quarter, viz., 947, took place within the 5th year of life—a startling mortality, which deserves a far more careful attention from the profession, than it has hitherto received, in order, if practicable, to lesson the susceptibility of the system to disease and death, in the earliest periods of existence, when the tenure of life is so feeble.

The number of deaths, from reported diseases alone, amounts to 1692. Other outlets to human life are recorded, as usual, under the heads of Still Born, Old Age, Debility, Unknown, and External Causes; in all, 384.

The excess of deaths among the male population has been 208, equal to 10 per cent. of the whole number. This precedence of death in the males, is, without doubt, occasioned by their greater exposure to the causes of disease.

One half the whole number of deaths from reported diseases are accredited to Consumption of the Lungs, Convulsions, Croup, Inflammation of the Lungs, Marasmus, Inflammation of the Bronchia, Scarlet and Typhoid Fevers.

Class 1st.—"Endemic and Contagious diseases." Under this distinction, there has taken place 422 deaths; a reduction of 58 per cent. below the third quarter's returns. This sensible falling off, has been brought about by the almost entire absence of Cholera Infantum and Dysentery, and the decline of Fevers—there being in these, alone, a difference of 567 in favor of the 4th quarter. Croup, however, appears to have prevailed to a considerable extent, if we judge from the number of deaths, amounting to 109. The deaths from Scarlet Fever, have also been on the progressive,

Class 2d.—The "Sporadic diseases." The deaths from this Class, present a favorable aspect, and show a decline, since the preceding quarter, of 113. Marasmus is charged with only 80 deaths, while the former quarter gives 145.

Class 3d.—"Nervous System." Here, also, we find a diminution in the mortality, equal to 200, since the last quarter's report. Convulsions, one of the common outlets of infantile existence, as usual, makes up more than one third the whole number of deaths from this class of diseases.

Class 4th.—"Organs of Respiration." As might be expected on the approach of cold weather, there has been an increase of fatality from this class of diseases, embracing, as it does, those inflammatory affections prevalent during our winter months. Consumption of the Lungs, Inflammation of the Lungs, and Inflammation of the Bronchia, stand conspicuously forth in this department of disease; amounting, in the aggregate, to 463, out of 549, the number charged to this class, or 86 per cent. of the whole.

Table No. 3 furnishes the number of deaths at fifteen distinct periods of life. As all former vital statistics have exhibited a marked mortality in infantile life over those of the other decades, so, in this instance, the first ten years present a large fatality, amounting to 1042, more than fifty per cent. of the whole number of deaths for the quarter.

The Blockley Alms House has furnished 146 of the deaths. The Blacks 114, while 19 were brought from a distance and added to our city mortality.

### TABLE No. I.

*Deaths for the Fourth Quarter of 1853, classified.*

| | October. | November. | December. | MALE. | | | FEMALE. | | | Total. |
|---|---|---|---|---|---|---|---|---|---|---|
| | | | | O. | N. | D. | O. | N. | D. | |
| 1. *Endemic and Contagious Diseases.* Zymotic or Epidemic . . | 164 | 124 | 134 | 92 | 72 | 67 | 72 | 52 | 67 | 422 |
| 2. *Uncertain or general seat.* Sporadic diseases . . . . | 113 | 95 | 79 | 59 | 51 | 40 | 54 | 44 | 39 | 287 |
| 3. Nervous System . . . | 77 | 114 | 117 | 46 | 69 | 64 | 31 | 45 | 53 | 308 |
| 4. Organs of Respiration . . . | 138 | 220 | 191 | 77 | 109 | 98 | 61 | 111 | 93 | 549 |
| 5. " Circulation . . . | 13 | 18 | 14 | 7 | 11 | 9 | 6 | 7 | 5 | 45 |
| 6. Digestive Organs . . . | 49 | 46 | 36 | 22 | 22 | 17 | 27 | 24 | 19 | 131 |
| 7. Urinary Organs . . . | 2 | 3 | 3 | 2 | 2 | 3 | | 1 | | 8 |
| 8. Organs of Generation . . | 6 | 4 | 3 | | | | 6 | 4 | 3 | 13 |
| 9. " Locomotion . . | 6 | 1 | 2 | 4 | 1 | | 2 | | 2 | 9 |
| 10. Integumentary System . . | | 2 | | | 1 | | | 1 | | 2 |
| 11. Old Age . . . . . . | 11 | 10 | 14 | 5 | 2 | 9 | 6 | 8 | 5 | 35 |
| 12. External Causes . . . . | 39 | 38 | 26 | 29 | 29 | 22 | 10 | 9 | 4 | 103 |
| Still Born . . . . . | 37 | 36 | 44 | 19 | 24 | 25 | 18 | 12 | 19 | 117 |
| Unknown . . . . . . | 13 | 15 | 19 | 8 | 12 | 13 | 5 | 3 | 6 | 4[illegible] |
| | 668 | 726 | 682 | 370 | 405 | 367 | 298 | 321 | 315 | 2076 |

### TABLE No. II.

1. *Endemic and Contagious Diseases—Zymotic or Epidemic.*

| | Male. | Female. | Under 1 yr. | 1 to 2. | 2 to 5. | 5 to 10. | 10 to 15. | 15 to 20. | 20 to 30. | 30 to 40. | 40 to 50. | 50 to 60. | 60 to 70. | 70 to 80. | 80 to 90. | 90 to 100. | 100 to 110. | Total. |
|---|---|---|---|---|---|---|---|---|---|---|---|---|---|---|---|---|---|---|
| Cholera Infantum . | 13 | 9 | 15 | 4 | 3 | | | | | | | | | | | | | 22 |
| Croup . . . . | 56 | 53 | 13 | 20 | 56 | 20 | | | | | | | | | | | | 109 |
| Diarrhœa . . . | 14 | 7 | 5 | 5 | 1 | | | | 2 | 3 | 2 | | 2 | | 1 | | | 21 |
| Dysentery . . . | 25 | 24 | 6 | 9 | 6 | 2 | | 1 | 2 | 3 | 8 | 5 | 5 | 1 | 1 | | | 49 |
| Erysipelas . . . | 10 | 4 | 6 | | | | | | 2 | 2 | 1 | | 1 | 2 | | | | 14 |
| Fever . . . | 3 | 1 | | 1 | | | | 1 | 1 | | | | | 1 | | | | 4 |
| " Bilious . . | 5 | 5 | | 1 | | 1 | 1 | | | 3 | 1 | | 1 | 1 | 1 | | | 10 |
| " " Malignant . | 2 | 1 | | | | | | | | 2 | | 1 | | | | | | 3 |
| " " Typhoid . | 1 | | | | | | | | | | | 1 | | | | | | 1 |
| " " Remittent . | 1 | 1 | | | | | | 1 | | | | 1 | | | | | | 2 |
| " Congestive . | 3 | 2 | | | 1 | 1 | | 1 | 1 | 1 | | | | | | | | 5 |
| " Intermittent . | 3 | 1 | 1 | | | | | | 2 | | 1 | | | | | | | 4 |
| " Nervous . . | | 1 | | | | | | | | | | | | 1 | | | | 1 |
| " Pernicious . . | | 1 | | | | | | | | | | | 1 | | | | | 1 |
| " Remittent . . | 4 | 2 | | 1 | 1 | 2 | | | | 1 | | | 1 | | | | | 6 |
| " Scarlet . . | 29 | 30 | 5 | 9 | 31 | 13 | 1 | | | | | | | | | | | 59 |
| " Typhus . . | 16 | 14 | | | | 1 | 3 | 3 | 10 | 6 | 4 | 3 | | | | | | 30 |
| " " Icterodes . | 2 | 1 | | | | | | | 1 | | 1 | 1 | | | | | | 3 |
| " " Remittent . | | 1 | | | | | | 1 | | | | | | | | | | 1 |
| " Typhoid . . | 33 | 21 | 2 | | 2 | 4 | 3 | 7 | 12 | 13 | 3 | 6 | 2 | | | | | 54 |
| Hooping Cough . . | 8 | 8 | 5 | 5 | 6 | | | | | | | | | | | | | 16 |
| Measles . . . | | 3 | | 1 | 2 | | | | | | | | | | | | | 3 |
| Small Pox . . . | 1 | | | | | | | | | 1 | | | | | | | | 1 |
| Syphilis . . . | | 1 | | | | | | | | 1 | | | | | | | | 1 |
| " Secondary . . | 1 | | 1 | | | | | | | | | | | | | | | 1 |
| Varicella . . . | 1 | | 1 | | | | | | | | | | | | | | | 1 |
| | 231 | 191 | 60 | 56 | 109 | 44 | 8 | 15 | 33 | 36 | 21 | 18 | 13 | 6 | 3 | | | 422 |

2. *Uncertain or General Seat.—Sporadic Diseases.*

| | Male. | Female. | Under 1 yr. | 1 to 2. | 2 to 5. | 5 to 10. | 10 to 15. | 15 to 20. | 20 to 30. | 30 to 40. | 40 to 50. | 50 to 60. | 60 to 70. | 70 to 80. | 80 to 90. | 90 to 100. | 100 to 110. | Total. |
|---|---|---|---|---|---|---|---|---|---|---|---|---|---|---|---|---|---|---|
| Abscess . . . | 2 | 1 | | | 1 | | | | | 1 | | 1 | | | | | | 3 |
| " Hand . . | 1 | | | | | | | | | | | | | 1 | | | | 1 |
| Angina . . . | | 1 | | | | | | | | | | 1 | | | | | | 1 |
| Asthma Thymicum . | | 1 | 1 | | | | | | | | | | | | | | | 1 |
| Cachexia . . . | 1 | | | 1 | | | | | | | | | | | | | | 1 |
| Cancer . . . | 1 | 3 | | | | | | | 1 | | | 3 | | | | | | 4 |
| " Breast . . | | 3 | | | | | | | | 1 | | 1 | 1 | | | | | 3 |
| " Neck . . | | 1 | | | | | | | | 1 | | | | | | | | 1 |
| Cyanosis . . . | 2 | 3 | 5 | | | | | | | | | | | | | | | 5 |
| Debility . . . | 37 | 45 | 34 | 3 | | 1 | | 1 | 4 | 1 | 6 | 5 | 11 | 12 | 3 | 1 | | 82 |
| Disease Malignant . | 1 | | | | | | | | | | | | 1 | | | | | 1 |
| Dropsy . . . | 21 | 20 | 3 | 2 | 5 | 2 | 1 | 1 | 7 | 7 | 4 | 3 | 3 | 3 | | | | 41 |
| Gangrene . . . | 1 | | | | | | | | | | | | 1 | | | | | 1 |
| " Mouth . . | 1 | | | | 1 | | | | | | | | | | | | | 1 |
| Gout . . . . | 1 | | | | | | | | | | | 1 | | | | | | 1 |
| Hemorrhage . . | 6 | 3 | 1 | | | | | | | 4 | 3 | 1 | | | | | | 9 |
| Inanition . . . | 10 | 9 | 14 | | 1 | | | | | 2 | | 1 | 1 | | | | | 19 |
| Inflammation . . | 2 | 2 | | | 1 | 1 | | 1 | 1 | | | | | | | | | 4 |
| Malformation . . | 3 | 1 | 4 | | | | | | | | | | | | | | | 4 |
| Marasmus . . . | 46 | 34 | 51 | 17 | 6 | | | | 1 | 1 | | 2 | | 2 | | | | 80 |
| Mortification . . | | 2 | | | | | | | | | | | 1 | 1 | | | | 2 |
| Scirrhus . . . | | 1 | | | | | | | 1 | | | | | | | | | 1 |
| Scrofula . . . | 6 | 2 | 3 | 2 | | | | 1 | | 2 | | | | | | | | 8 |
| Sore Throat . . | 1 | | | | | 1 | | | | | | | | | | | | 1 |
| Stricture . . . | 1 | | | | | | | | | | 1 | | | | | | | 1 |
| Tabes Mesenterica . | 6 | 1 | 3 | 2 | | 2 | | | | | | | | | | | | 7 |
| Tumor, . . . | | 1 | | | | | | | | | | 1 | | | | | | 1 |
| Ulceration, . . . | | 1 | | | 1 | | | | | | | | | | | | | 1 |
| " Throat . . | | 2 | | | | 1 | | | | | | | 1 | | | | | 2 |
| | 150 | 137 | 119 | 27 | 16 | 8 | 1 | 4 | 15 | 20 | 14 | 20 | 20 | 19 | 3 | 1 | | 287 |

3. *Of the Nervous System.*

| | Male. | Female. | Under 1 yr. | 1 to 2. | 2 to 5. | 5 to 10. | 10 to 15. | 15 to 20. | 20 to 30. | 30 to 40. | 40 to 50. | 50 to 60. | 60 to 70. | 70 to 80. | 80 to 90. | 90 to 100. | 100 to 110. | Total. |
|---|---|---|---|---|---|---|---|---|---|---|---|---|---|---|---|---|---|---|
| Abscess of Brain . . | | 1 | 1 | | | | | | | | | | | | | | | 1 |
| Apoplexy . . . | 15 | 11 | 2 | | | | | | 1 | 4 | 4 | 5 | 5 | 1 | 4 | | | 26 |
| Concussion of Brain . | | 1 | | 1 | | | | | | | | | | | | | | 1 |
| Congestion " . | 14 | 19 | 11 | 9 | 3 | | 2 | | 2 | | 2 | 2 | | 1 | 1 | | | 33 |
| Convulsions . . | 65 | 47 | 59 | 22 | 19 | 4 | | 1 | 4 | | 3 | | | | | | | 112 |
| Disease of Brain . . | 8 | 5 | 4 | 1 | 5 | | | | 1 | 1 | | | 1 | | | | | 13 |
| Dropsy of Brain . . | 26 | 13 | 14 | 10 | 8 | 2 | 1 | | 3 | | | | 1 | | | | | 39 |
| Effusion " . . | 7 | 7 | 5 | 2 | 5 | 1 | | | | 1 | | | | | | | | 14 |
| Epilepsy . . . | 5 | 2 | 3 | | | | | | | | 2 | 1 | | 1 | | | | 7 |
| Inflammation of Brain . | 21 | 9 | 10 | 2 | 5 | 5 | 2 | | 2 | 2 | 1 | | | 1 | | | | 30 |
| Mania . . . | 2 | 3 | | | | | | | 1 | 2 | 1 | | 1 | | | | | 5 |
| " a potu . . | 8 | | | | | | | | 1 | 2 | 4 | 1 | | | | | | 8 |
| Palsy . . . . | 6 | 6 | | | | | | | 1 | 3 | 1 | 2 | 2 | 2 | 1 | | | 12 |
| Softening of Brain . | 2 | 4 | 1 | | | | | | 1 | | | 1 | | 2 | 1 | | | 6 |
| Tetanus . . . | | 1 | | | | | | | | | 1 | | | | | | | 1 |
| | 179 | 129 | 110 | 47 | 45 | 12 | 5 | 1 | 17 | 15 | 19 | 12 | 10 | 8 | 7 | | | 3 |

### 4. *Organs of Respiration.*

| | Male. | Female. | Under 1 yr. | 1 to 2. | 2 to 5. | 5 to 10. | 10 to 15. | 15 to 20. | 20 to 30. | 30 to 40. | 40 to 50. | 50 to 60. | 60 to 70. | 70 to 80. | 80 to 90. | 90 to 100. | 100 to 110. | Total. |
|---|---|---|---|---|---|---|---|---|---|---|---|---|---|---|---|---|---|---|
| Asthma . . . | 2 | 2 | 2 | | | | | | | | 1 | 1 | | | | | | 4 |
| Congestion of Lungs . | 8 | 7 | 3 | 3 | | | | 1 | 1 | 2 | 2 | 1 | 1 | 1 | | | | 15 |
| Consumption " . | 152 | 156 | 5 | 6 | 5 | 3 | 8 | 20 | 106 | 71 | 32 | 30 | 15 | 5 | 2 | | | 308 |
| Disease of Chest . . | 2 | 1 | 1 | 1 | 1 | | | | | | | | | | | | | 3 |
| " Lungs . | 6 | 5 | 3 | 1 | | 1 | | 2 | 1 | | 2 | | | 1 | | | | 11 |
| Dropsy of Chest . . | 3 | 7 | | 1 | | 1 | | | | | 2 | 2 | 3 | 1 | | | | 10 |
| Edema of Lungs . | 1 | 1 | | | | 1 | | | | | | | 1 | | | | | 2 |
| Effusion " . . | 1 | | | | | 1 | | | | | | | | | | | | 1 |
| " of Chest . . | 3 | | 1 | | 2 | | | | | | | | | | | | | 3 |
| Emphysema of Lungs . | 1 | | | | | | | | | 1 | | | | | | | | 1 |
| Fever, hectic . . | 1 | | 1 | | | | | | | | | | | | | | | 1 |
| Gangrene of Lungs . | 1 | | | | | | | | | 1 | | | | | | | | 1 |
| Hemorrhage of Lungs . | 4 | 2 | | | | | | | 4 | 1 | | | | | | | | 6 |
| Inflamm'n of Bronchiæ . | 40 | 25 | 24 | 19 | 5 | 3 | | | 1 | 1 | 2 | 2 | 3 | 4 | 1 | | | 65 |
| " Chest . | 1 | 3 | 1 | 3 | | | | | | | | | | | | | | 4 |
| " Fauces . | 1 | | 1 | | | | | | | | | | | | | | | 1 |
| " Larynx . | 1 | 1 | | | 2 | | | | | | | | | | | | | 2 |
| " Lungs . | 47 | 43 | 27 | 10 | 12 | 5 | 2 | 1 | 6 | 3 | 10 | 5 | 7 | 1 | 1 | | | 90 |
| " Pleura . | 5 | 2 | | | | 1 | 1 | | 2 | 1 | 1 | | 1 | | | | | 7 |
| " Tonsils . | | 2 | | | 1 | | | | | 1 | | | | | | | | 2 |
| Tuberculosis . . | 2 | 6 | | 1 | 1 | | | 1 | 2 | 2 | | 1 | | | | | | 8 |
| Ulceration of Larynx . | 1 | | 1 | | | | | | | | | | | | | | | 1 |
| " Lungs . | 1 | 1 | | | | | | 1 | | 1 | | | | | | | | 2 |
| " Trachea . | | 1 | | | 1 | | | | | | | | | | | | | 1 |
| | 284 | 265 | 70 | 46 | 30 | 16 | 11 | 26 | 123 | 85 | 52 | 42 | 31 | 13 | 4 | | | 549 |

### 5. *Organs of Circulation.*

| | Male. | Female. | Under 1 yr. | 1 to 2. | 2 to 5. | 5 to 10. | 10 to 15. | 15 to 20. | 20 to 30. | 30 to 40. | 40 to 50. | 50 to 60. | 60 to 70. | 70 to 80. | 80 to 90. | 90 to 100. | 100 to 110. | Total. |
|---|---|---|---|---|---|---|---|---|---|---|---|---|---|---|---|---|---|---|
| Anemia . . . | | 1 | | | | | | | | 1 | | | | | | | | 1 |
| Aneurism . . . | 1 | 1 | | | | | | | 2 | | | | | | | | | 2 |
| " of Aorta . | 1 | | | | | | | | | | 1 | | | | | | | 1 |
| Angina Pectoris . . | 1 | | | | | | | | | | | 1 | | | | | | 1 |
| Disease of Heart . . | 12 | 10 | 3 | | 1 | | 1 | | 5 | 1 | 2 | 2 | 4 | 1 | | 2 | | 22 |
| Dropsy " . . | 4 | 2 | | | 2 | | | | 2 | 1 | | | | 1 | | | | 6 |
| Enlargem't " . . | 3 | 3 | | | | | | 2 | 1 | 1 | 1 | 1 | | | | | | 6 |
| Gout " . . | 1 | | | | | | | | | | | 1 | | | | | | 1 |
| Inflammation " . . | 4 | 1 | | | | 1 | | | 1 | 2 | 1 | | | | | | | 5 |
| | 27 | 18 | 3 | | 3 | 1 | 1 | 2 | 11 | 6 | 5 | 5 | 4 | 2 | | 2 | | 45 |

### 8. *Organs of Generation.*

| | Male. | Female. | Under 1 yr. | 1 to 2. | 2 to 5. | 5 to 10. | 10 to 15. | 15 to 20. | 20 to 30. | 30 to 40. | 40 to 50. | 50 to 60. | 60 to 70. | 70 to 80. | 80 to 90. | 90 to 100. | 100 to 110. | Total. |
|---|---|---|---|---|---|---|---|---|---|---|---|---|---|---|---|---|---|---|
| Cancer of Uterus . . | | 4 | | | | | | | | | 2 | 1 | 1 | | | | | 4 |
| Child Bed . . . | | 1 | | | | | | | | 1 | | | | | | | | 1 |
| Hemorrhage of Uterus. | | 1 | | | | | | | | | 1 | | | | | | | 1 |
| Inflammation " . | | 1 | | | | | | | | 1 | | | | | | | | 1 |
| Puerperal Convulsions. | | 3 | | | | | | | 2 | 1 | | | | | | | | 3 |
| " Fever . | | 2 | | | | | | | | 2 | | | | | | | | 2 |
| Rupture of Uterus . | | 1 | | | | | | | | 1 | | | | | | | | 1 |
| | | 13 | | | | | | | 2 | 6 | 3 | 1 | 1 | | | | | 13 |

### 6. *Digestive Organs.*

| | Male. | Female. | Under 1 yr. | 1 to 2. | 2 to 5. | 5 to 10. | 10 to 15. | 15 to 20. | 20 to 30. | 30 to 40. | 40 to 50. | 50 to 60. | 60 to 70. | 70 to 80. | 80 to 90. | 90 to 100. | 100 to 110. | Total. |
|---|---|---|---|---|---|---|---|---|---|---|---|---|---|---|---|---|---|---|
| Abcess of Liver . | 1 | | | | | | | | | | 1 | | | | | | | 1 |
| " Rectum . | 1 | | 1 | | | | | | | | | | | | | | | 1 |
| Bilious Colic . . | 1 | | | | | | | | 1 | | | | | | | | | 1 |
| Cancer of Stomach . | 2 | 1 | | | | | | | | | 1 | 2 | | | | | | 1 |
| " Rectum . | | 1 | | | | | | | | | | | 1 | | | | | 3 |
| Cancrum Oris . . | | 2 | 2 | | | | | | | | | | | | | | | 1 |
| Consumption of Bowels | 2 | | | | 2 | | | | | | | | | | | | | 2 |
| Colic . . . . . | 1 | | | | | | | | 1 | | | | | | | | | 2 |
| Disease of Bowels . | 1 | 1 | | | | | | | | | 1 | | | 1 | | | | 1 |
| " Liver . . | 1 | 2 | | | | | | | | | 1 | 1 | 1 | | | | | 3 |
| " Stomach . | 2 | 2 | | | | | | | 1 | | 2 | | | 1 | | | | 4 |
| Dropsy Abdominal . . | 4 | 2 | | | | | | | 1 | | 1 | 1 | 1 | 1 | 1 | | | 6 |
| Enlargement of Liver . | 1 | | | | | | | | | 1 | | | | | | | | 1 |
| Fatty degeneration " . | | 1 | | | | | | | 1 | | | | | | | | | 1 |
| Gangrenopsis . . | | 1 | | | | | | | 1 | | | | | | | | | 1 |
| Hemorrhage of Stom'h . | 1 | 2 | | | | | 1 | | | | 1 | 1 | | | | | | 3 |
| Ileus . . . . . | | 1 | | | | | | | | | 1 | | | | | | | 1 |
| Inflam'n of Sto'h & Bow'ls | 31 | 27 | 15 | 14 | 5 | 2 | 3 | 2 | 6 | | 2 | 4 | 2 | 2 | 1 | | | 58 |
| " Liver . . | 2 | 4 | | | | | | | | 3 | 1 | 1 | | 1 | | | | 6 |
| " Peritoneum. | 1 | 5 | | | | | | | | 3 | 1 | 1 | 1 | | | | | 6 |
| Jaundice . . . | 2 | 4 | 2 | | 1 | | | | | 1 | 1 | | 1 | | | | | 6 |
| Mortification of Bowels | | 1 | | | | | | | 1 | | | | | | | | | 1 |
| Obstruction " . | 1 | 1 | | | | | | | | 1 | | | | 1 | | | | 2 |
| Perforation " . | 1 | | | | | | | | 1 | | | | | | | | | 1 |
| Scirrhus of Liver . | | 2 | | | | | | | | 1 | 1 | | | | | | | 2 |
| Softening of Stomach . | 1 | | 1 | | | | | | | | | | | | | | | 1 |
| Strangulated Hernia . | | 1 | | | | | | | | | | | 1 | | | | | 1 |
| Stricture of Esophagus. | 1 | | | | | | | | | | | | 1 | | | | | 1 |
| Teething . . . | | 3 | 1 | 1 | 1 | | | | | | | | | | | | | 3 |
| Tub'r affection of Abdom. | | 1 | | | | | | | | | | | 1 | | | | | 1 |
| Ulceration of Bowels . | 3 | 4 | | 1 | | 1 | | | 1 | 2 | 2 | | | | | | | 7 |
| Worms . . . | | 1 | | 1 | | | | | | | | | | | | | | 1 |
| | 61 | 70 | 22 | 17 | 9 | 3 | 4 | 2 | 15 | 12 | 17 | 11 | 10 | 7 | 2 | | | 131 |

7. *The Urinary Organs.*

| | Male. | Female. | Under 1 yr. | 1 to 2. | 2 to 5. | 5 to 10. | 10 to 15. | 15 to 20. | 20 to 30. | 30 to 40. | 40 to 50. | 50 to 60. | 60 to 70. | 70 to 80. | 80 to 90. | 90 to 100. | 100 to 110. | Total. |
|---|---|---|---|---|---|---|---|---|---|---|---|---|---|---|---|---|---|---|
| Diabetes . . | 1 | | | | | | | | | | 1 | | | | | | | 1 |
| Dissease of Kidneys . | 2 | | | | | | | | | 2 | | | | | | | | 2 |
| " of Urinary Organs | | 1 | | | | | | | | | | 1 | | | | | | 1 |
| Inflam. of Bladder . | 3 | | | | | | | | | | 1 | 1 | | 1 | | | | 3 |
| " Kidneys . | 1 | | | | | | | | | 1 | | | | | | | | 1 |
| | 7 | 1 | | | | | | | | 3 | 2 | 2 | | 1 | | | | 8 |

9. *Organs of Locomotion.*

| | Male. | Female. | Under 1 yr. | 1 to 2. | 2 to 5. | 5 to 10. | 10 to 15. | 15 to 20. | 20 to 30. | 30 to 40. | 40 to 50. | 50 to 60. | 60 to 70. | 70 to 80. | 80 to 90. | 90 to 100. | 100 to 110. | Total. |
|---|---|---|---|---|---|---|---|---|---|---|---|---|---|---|---|---|---|---|
| Abscess of Knee Joint . | 1 | | | | 1 | | | | | | | | | | | | | 1 |
| Caries of Femur . . | 1 | | | | | | | | | 1 | | | | | | | | 1 |
| " Spine . . | | 1 | | | 1 | | | | | | | | | | | | | 1 |
| Disease of " . . | | 1 | | | | | | 1 | | | | | | | | | | 1 |
| Gangrene of Foot . | 1 | | | | | | | | | | 1 | | | | | | | 1 |
| Inflammation of Spine . | 1 | | | | | | | 1 | | | | | | | | | | 1 |
| Injury of " . | 1 | | | | | | | | 1 | | | | | | | | | 1 |
| Rheumatism . . | | 1 | | | | | | | | | 1 | | | | | | | 1 |
| Spina Bifida . . | | 1 | 1 | | | | | | | | | | | | | | | 1 |
| | 5 | 4 | 1 | | 2 | | | 2 | 1 | 1 | 2 | | | | | | | 9 |

10. *The Integumentary System.*

| | Male. | Female. | Under 1 yr. | 1 to 2. | 2 to 5. | 5 to 10. | 10 to 15. | 15 to 20. | 20 to 30. | 30 to 40. | 40 to 50. | 50 to 60. | 60 to 70. | 70 to 80. | 80 to 90. | 90 to 100. | 100 to 110. | Total. |
|---|---|---|---|---|---|---|---|---|---|---|---|---|---|---|---|---|---|---|
| Disease of Skin . . | 1 | | 1 | | | | | | | | | | | | | | | 1 |
| Purpura Hemorrhagica | | 1 | | | 1 | | | | | | | | | | | | | 1 |
| | 1 | 1 | 1 | | 1 | | | | | | | | | | | | | 2 |

11. *Old Age.*

| | Male. | Female. | Under 1 yr. | 1 to 2. | 2 to 5. | 5 to 10. | 10 to 15. | 15 to 20. | 20 to 30. | 30 to 40. | 40 to 50. | 50 to 60. | 60 to 70. | 70 to 80. | 80 to 90. | 90 to 100. | 100 to 110. | Total. |
|---|---|---|---|---|---|---|---|---|---|---|---|---|---|---|---|---|---|---|
| Old Age . . . | 16 | 19 | | | | | | | | | | | 1 | 13 | 14 | 7 | | 35 |

12. *From External Causes.*

| | Male. | Female. | Under 1 yr. | 1 to 2. | 2 to 5. | 5 to 10. | 10 to 15. | 15 to 20. | 20 to 30. | 30 to 40. | 40 to 50. | 50 to 60. | 60 to 70. | 70 to 80. | 80 to 90. | 90 to 100. | 100 to 110. | Total. |
|---|---|---|---|---|---|---|---|---|---|---|---|---|---|---|---|---|---|---|
| Asphyxia . . . | 5 | 2 | 6 | | | | | | | | 1 | | | | | | | 7 |
| Burns . . . | 9 | 8 | 2 | 2 | 5 | 3 | | 1 | 1 | 1 | | 1 | 1 | | | | | 17 |
| Casualties . . . | 29 | 4 | 1 | | 2 | 3 | 2 | 1 | 11 | 5 | 2 | 3 | 2 | 1 | | | | 33 |
| Drowned . . . | 17 | | | | | 1 | | 1 | 9 | 3 | 2 | | 1 | | | | | 17 |
| Fracture . . . | 2 | | | 1 | | 1 | | | | | | | | | | | | 2 |
| " of Skull . . | 1 | | | | 1 | | | | | | | | | | | | | 1 |
| " of Spine . . | 1 | | | | | | | | 1 | | | | | | | | | 1 |
| Intemperance . . | 13 | 7 | | | | | | 1 | 3 | 9 | 4 | 2 | 1 | | | | | 20 |
| Poisoning . . . | 1 | | | | | 1 | | | | | | | | | | | | 1 |
| Strangulation . . | 1 | 2 | 1 | | 1 | | | | | | | 1 | | | | | | 3 |
| Suffocation . . . | 1 | | | 1 | | | | | | | | | | | | | | 1 |
| | 80 | 23 | 10 | 4 | 9 | 9 | 2 | 4 | 25 | 18 | 9 | 7 | 5 | 1 | | | | 103 |
| Still Born . . . | 68 | 49 | 117 | | | | | | | | | | | | | | | 117 |
| Unknown . . . | 33 | 14 | 8 | 1 | 4 | 2 | | 2 | 5 | 6 | 10 | 5 | 4 | | | | | 47 |

TABLE NO. 3.

*Deaths for the Fourth Quarter of* 1853, *at fifteen distinct periods of life.*

| | |
|---|---|
| Under 1 year, . . . . . . | 404 |
| 1 to 2 . . . . . . . | 198 |
| 2 to 5 . . . . . . . | 228 |
| 5 to 10 . . . . . . . | 95 |
| 10 to 15 . . . . . . . | 32 |
| 15 to 20 . . . . . . . | 58 |
| 20 to 30 . . . . . . . | 247 |
| 30 to 40 . . . . . . . | 208 |
| 40 to 50 . . . . . . . | 154 |
| 50 to 60 . . . . . . | 123 |
| 60 to 70 . . . . . . . | 99 |
| 70 to 80 . . . . . . . | 70 |
| 80 to 90 . . . . . . . | 33 |
| 90 to 100 . . . . . . . | 10 |
| 100 to 110 . . . . . . . | 0 |
| | 1959 |
| Still Born . . . . . . . | 117 |
| Total, | 2076 |

Embraced within the foregoing tables, were 146 from the Blockley Alms House, 114 persons of color, and 19 from the country, as follows:

| | October. | November. | December. | Total. |
|---|---|---|---|---|
| Almshouse, . . | 61 | 51 | 34 | 146 |
| Blacks, . . . | 40 | 43 | 31 | 114 |
| Country, . . | 7 | 7 | 5 | 19 |
| Total, | | | | 279 |

### SUMMARY OF DEATHS FOR THE WHOLE YEAR.

In preparing a general summary for 1853 we find the deaths, from all causes, to be 9744, a decrease under those of 1852 of 514, or 5 pr. ct., while it is well understood that our population has increased. This is an indication of the generally improved health of the city during the past year. The excess of deaths in 1852 over those for 1851, amounted to 13 pr. ct.

Estimating the population to be 415,000 the aggregate of deaths will give one in every 42½.

The average of deaths for each month is 812; for each week, 187½ and for each day 26½.

The highest mortality occurred in August, viz. 1235, equal to 40 deaths per day. The lowest was in May, 597. This augmentation of mortality in August, may be charged to convulsions, cholera infantum, dysentery and yellow fever, which latter disease prevailed in the vicinity of South street wharf.

While the total of deaths for the year has been 9744, the actual number who have died from accredited disease was only 8026. This may be explained by deducting 1718 deaths placed under the head of unknown, still born, external causes, old age, malformation and debility.

The deaths among the adult population, or those over 20 years amounted to 4697. Of children, or those under twenty, 5047.

During the first few years of life the rate of mortality is very high; 2150, or 22 pr. ct. of all the deaths took place within the first year. 4153, or 43 pr. ct. occurred before the fifth year. In the tenth decade of life, or over ninety, there were 38 deaths recorded, and 2 over one hundred years of age. These calculations include the still born, which amounted to 565.

The subjoined statement shows the total of deaths for 1853 in their classified order.

| | Males. | Females. | Total. |
|---|---|---|---|
| 1. *Endemic and contagious diseases,* | | | |
| Zymotic or Epidemic, . . . . . | 1287 | 1135 | 2422 |
| 2. *Uncertain, or General Seat,* | | | |
| Sporadic diseases, . . . . . . | 677 | 622 | 1299 |
| 3. Nervous system, . . . . | 917 | 697 | 1614 |
| 4. Organs of Respiration, . . . . | 1020 | 1057 | 2077 |
| 5. " Circulation, . . . . . . | 102 | 107 | 209 |
| 6. " Digestion, . . . . . | 310 | 299 | 609 |
| 7. " Urinary, . . . . . . | 30 | 10 | 40 |
| 8. " Generation, . . . . | 2 | 99 | 101 |
| 9. " Locomotion, . . . . . | 20 | 25 | 45 |
| 10. Integumentary system, . . . . | 13 | 7 | 20 |
| 11. Old Age, . . . . . . | 54 | 79 | 133 |
| 12. External Causes, . . . . . | 277 | 119 | 396 |
| Still Born, . . . . . . . | 324 | 241 | 565 |
| Unknown, . . . . . . . | 132 | 82 | 214 |
| | 5165 | 4579 | 9744 |

By this analytical arrangement, we learn at a single glance the mortality from diseases, epidemic or sporadic in their character, or those implicating particular organs of the body, or those from external and other causes for the termination of life.

As in former vital statistics, this table makes the excess of deaths on the side of the males; equal this year to 11 pr. ct. In 1852 it was 13 per cent. In the "still born," the excess of males is 26 per ct.; in 1852 it was 30 per ct.

The deaths from the classes "zymotic," "sporadic," "nervous system" and the "organs of respiration" hold a conspicuous place in the above table. The first contributes 25 pr. ct.; the second, 13 pr. ct.; the third, or nervous system, 16 pr. ct., and the organs of respiration 21 pr. ct. of the mortality.

Consumption of the lungs numbers 1246 among its victims, being an increase of 3½ pr. ct. above those of 1852. This large amount exceeds by far those of any other disease in the catalogue, and gives one out of every 7¾ deaths during the year. Equal to 12 pr. ct. of the whole number. It does not appear to confine its ravages to any one particular period of the year, nor is any decade of life exempt from its attack. According to the record, both infancy and old age come within its range. Seventeen deaths under one year of age are charged to its account, while four, over eighty, have been caused by its resistless power. As heretofore observed, it seeks its prey at the most interesting and favorable period of human existence, between twenty and thirty.

During 1853, it carried off in this decade 403, nearly one third of the whole number recorded. The excess of deaths was among females.

The annexed table furnishes a synopsis of the diseases which have been most fatal, and hence most prevalent for six consecutive years in this city.

| | 1848. | 1849. | 1850. | 1851. | 1852. | 1853. |
|---|---|---|---|---|---|---|
| Cholera Infantum, | 454 | 582 | 505 | 397 | 329 | 398 |
| Consumption of the Lungs, | 965 | 939 | 907 | 881 | 1204 | 1246 |
| Congestion of the Brain, | 85 | 91 | 97 | 130 | 120 | 172 |
| Convulsions, | 401 | 415 | 444 | 479 | 499 | 543 |
| Croup, | 177 | 130 | 143 | 180 | 208 | 303 |
| Diarrhœa, | 122 | 225 | 208 | 157 | 156 | 130 |
| Dropsy of the Brain, | 220 | 237 | 283 | 245 | 247 | 192 |
| Dysentery, | 315 | 578 | 421 | 401 | 558 | 379 |
| Inflammation of the Brain, | 186 | 198 | 218 | 202 | 258 | 157 |
| " Bronchiæ, | 172 | 169 | 191 | 175 | 208 | 197 |
| " Lungs, | 265 | 273 | 352 | 352 | 444 | 353 |
| Marasmus, | 237 | 264 | 217 | 255 | 354 | 376 |
| Scarlet Fever, | 172 | 242 | 439 | 400 | 433 | 391 |
| Small Pox, | 100 | 152 | 40 | 216 | 426 | 52 |

